# A light-controlled one-tube detection platform combining CRISPR-Cas12a and RPA: an innovative approach for rapid diagnosis of *Acinetobacter baumannii*

**DOI:** 10.3389/fbioe.2025.1663915

**Published:** 2025-08-11

**Authors:** Zihan Zhou, Lele Pan, Shihua Luo, Jiangmei Ma, Baoyan Ren, Lina Liang, Xuebin Li, Guijiang Wei

**Affiliations:** ^1^ Center for Medical Laboratory Science, Affiliated Hospital of Youjiang Medical University for Nationalities, Guangxi, Baise, China; ^2^ Key Laboratory of Research on Clinical Molecular Diagnosis for High Incidence Diseases in Western Guangxi of Guangxi Higher Education Institutions, Guangxi, Baise, China; ^3^ Baise Key Laboratory for Precise Genetic Testing of Long-dwelling Nationalities, Guangxi, Baise, China; ^4^ Engineering Research Center of Guangxi Higher Education Institutions for Precise Genetic Testing of Long-dwelling Nationalities, Guangxi, Baise, China; ^5^ Guangxi Engineering Research Center for Precise Genetic Testing of Long-dwelling Nationalities, Guangxi, Baise, China; ^6^ Yaneng BlOscience (Shenzhen) Corporation, Guangdong, Shenzhen, China; ^7^ Modern Industrial College of Biomedicine and Great Health, Youjiang Medical University for Nationalities, Guangxi, Baise, China

**Keywords:** CRISPR/Cas12a, NPOM-dt, one-tube detection, POCT, *Acinetobacter* baumannii

## Abstract

**Background:**

*Acinetobacter baumannii (A. baumannii)* is a significant pathogen associated with nosocomial infections, predominantly affecting immunocompromised patients, and is linked to high mortality rates. To control infection rates, there is an urgent need for a diagnostic method that is cost-effective, rapid, and user-friendly, meeting the current demand for timely diagnosis.

**Methods:**

We have developed a one-tube detection method for UV light unlocking based on RPA-CRISPR/Cas12a technology. This method utilizes the photodegradable chemical group NPOM-dt to chemically modify the crRNA base, preventing it from complementary pairing with the base of the target molecule, thereby temporarily silencing the CRISPR system. After RPA preamplification, the caged modification group on the crRNA was removed with brief irradiation with ultraviolet light to restore the activity of the CRISPR/Cas12a. system.

**Results:**

Our results demonstrated that the detection system achieved a limit of detection as low as 10 copies/μL for target fragments, with no cross-reactivity observed with genomic DNA from six clinically common pathogenic bacteria, showcasing excellent sensitivity and specificity. Additionally, clinical validation was performed using 38 sputum samples. The system successfully identified A. baumannii in sputum specimens, with results consistent with those obtained via conventional PCR.

**Conclusion:**

We have successfully developed a light-controlled one-tube RPA-CRISPR/Cas12a detection system. It simplifies the operation and at the same time greatly reduces the risk of laboratory contamination caused by repeated tube opening, providing a new idea for the development of point-of-care testing (POCT).

## 1 Introduction

*Acinetobacter baumannii* (*A. baumannii*), a Gram-negative opportunistic pathogen, is ubiquitously found in the environment and serves as a major contributor to severe hospital-acquired infections. Currently, multidrug-resistant strains of *A. baumannii*, which pose a substantial threat to public health, are predominantly prevalent in hospital settings. This pathogen demonstrates remarkable resilience, surviving for extended periods on inanimate surfaces. It is primarily transmitted through the hospital environment, healthcare personnel, and medical equipment, earning it the designation of a “nosocomial superbug” (Srikanth et al., 2022). *A. baumannii* can inhabit the respiratory tract, urinary tract, and skin. In individuals with compromised immunity or skin injuries, it can quickly multiply and secrete bacterial toxins, leading to localized inflammatory responses at the infection site. This pathogen is associated with a variety of infections, including respiratory infections, bloodstream infections, urinary tract infections, intracranial infections, as well as skin and soft tissue infections (Xu et al., 2021). In recent years, the widespread clinical use of carbapenem antibiotics has led to an increasingly alarming rise in carbapenem resistance in *A*. *baumannii*. (Shi et al., 2024; Su et al., 2019; Jiang et al., 2022). Class D oxacillinases (OXAs) is unique to *Acinetobacter* and contains several isoforms, with OXA-23, OXA-24, OXA-51, and OXA-58 being the most prominent (Nordmann and Poirel, 2019; Wohlfarth et al., 2022). According to a study by Zhang Y et al., blaOXA-23 is the primary factor responsible for carbapenem resistance in *A. baumannii*. Research by Merkier et al. has shown that the blaOXA-51 gene is a key marker for the identification of *A. baumannii* (Zhang et al., 2021; Merkier and Centrón, 2006). *A. baumannii* poses a serious threat to human health and safety, making the rapid, efficient, and simple diagnosis of this pathogen critically important.

Currently, common methods for detecting *A. baumannii* include bacterial culture, mass spectrometry identification, and polymerase chain reaction (PCR). Bacterial culture, recognized as the gold standard for bacterial diagnosis, is widely accepted across healthcare institutions. While it is cost-effective, it has limitations such as long turnaround times (2–3 days) and cumbersome procedures. Mass spectrometry-based identification is straightforward, rapid, accurate, and high-throughput, but it requires the establishment of a microbial protein fingerprinting database in the cloud (Girard et al., 2021). PCR offers high sensitivity and specificity; however, it requires specialized equipment, strict thermal cycling conditions, high-quality reagents, skilled technicians, and a well-maintained laboratory environment. Due to the complexity of the equipment and the stringent environmental requirements, PCR is less feasible in grassroots hospitals with limited medical infrastructure. Recombinant polymerase amplification (RPA), as a representative of isothermal amplification technology, has attracted great attention in recent years. Unlike PCR, which involves multiple temperature gradients, RPA utilizes components such as recombinases to achieve rapid amplification of target genes at constant temperatures (typically between 37°C and 42°C), and the reaction is completed in approximately 20 min, providing significantly reduced turnaround time while maintaining excellent specificity and sensitivity (Wang et al., 2024; Munawar, 2022; Yang et al., 2020). By integrating downstream visualization technologies such as agarose gel electrophoresis and lateral flow test strips, a variety of results can be analyzed. As a potential alternative to PCR, RPA has great future use prospects.

The clustered regularly interspaced short palindromic repeats (CRISPR) system, along with its associated proteins (Cas), serves as an adaptive immune mechanism in certain archaea, providing defense against the intrusion of “non-self” elements (Koonin et al., 2017). In recent years, it has been widely applied in gene editing, single nucleotide polymorphisms, pathogen microbiology, exosome research, and cancer studies, offering novel insights and approaches for the advancement of molecular diagnostic technologies (Eisenstein, 2022). The CRISPR-Cas system, with its powerful single-base recognition ability and collateral cleavage activity, is frequently combined with various nucleic acid amplification techniques. Diagnostic strategies such as SHERLOCK and DETECTR have been developed and are now widely used in the development of point-of-care testing (POCT) kits (Di Carlo and Sorrentino, 2024; Gootenberg et al., 2017; Zhou et al., 2025). The combination of RPA and the CRISPR-Cas12a system is a commonly used strategy in targeted DNA molecular diagnostics, significantly enhancing both diagnostic specificity and sensitivity. This approach is comparable to mainstream qPCR methods.

However, most CRISPR-based nucleic acid diagnostic platforms currently developed involve multiple steps and spatial transfer, with target amplification and CRISPR detection performed in separate tubes. This complexity not only complicates the procedure but also increases the risk of cross-contamination (Li et al., 2023). While methods such as physical separation using gels, paraffin, mother-daughter tubes, or directly adding the CRISPR system to the tube cap have been proposed to enable single-tube operations, these approaches still require temperature fluctuations or manual centrifugation steps (Qiu et al., 2023; Uno et al., 2023; Tan et al., 2024; Bhardwaj et al., 2025).

Precise regulation of biochemical functions can be achieved by modifying photochemically removable motifs on primers or target sites. Photocaged moieties have been successfully applied to the regulation of gene expression (Deiters, 2009), enzyme activity (Young and Deiters, 2006) and RNA function (Chaulk and MacMillan, 1998). Recent study have suggested that the guide RNA in the CRISPR-Cas9 system can be cage-modified with chemical groups, which blocks its complementary pairing with the target sequence, temporarily silencing its activity. Upon exposure to 365 nm ultraviolet (UV) light, the cage modification is release, thereby restoring the activity of the complex (Wang et al., 2020; Liu et al., 2024). To detect *A. baumannii* during the infection window period and control the infection rate, we developed a novel one-tube detection method based on RPA-CRISPR/Cas12a technology, utilizing a photolysis unlocking approach. This method integrates RPA target amplification and CRISPR-specific signal amplification in a single tube. Initially, we chemically modified the crRNA to prevent its complementary pairing with the target molecule, thereby temporarily silencing the CRISPR system. After completing the RPA pre-amplification, UV light is used to induce the removal of the protective chemical group, restoring the activity of the CRISPR/Cas12a system. This photolysis unlocking one-tube detection strategy is more convenient and faster than the traditional two-step RPA-CRISPR method, and it also minimizes the risk of potential aerosol contamination caused by repeated tube opening.

## 2 Materials and methods

### 2.1 Reagents and sample

All reference strains utilized in this study were sourced from the strain repository maintained by the Medical Laboratory Department of the Affiliated Hospital of Youjiang Medical University for Nationalities. The strains included *A. baumannii* (ATCC 19606), *Escherichia coli* (ATCC 25922), *Stenotrophomonas maltophilia* (ATCC 17676), *Klebsiella pneumoniae* (ATCC 13883), *Pseudomonas aeruginosa* (ATCC 27853), *Staphylococcus aureus* (ATCC 25923), and *Haemophilus haemolyticus* (ATCC 33390). The OXA-51 gene fragment of *A. baumannii* (GenBank: KP462889.1) was cloned into the pUC19 vector by Sangon Biotechnology Co. (Shanghai, China) to construct the PUC19-51 plasmid, which served as the standard template for subsequent experiments. The concentration of PUC19-OXA-51 was determined using a NanoDrop ND-2000 spectrophotometer (NanoDrop Technologies, United States). Additionally, 38 clinical sputum samples were collected from patients by the Microbiology Laboratory of the Medical Laboratory Department at the Affiliated Hospital of Youjiang Medical University for Nationalities. The collection and use of these samples were approved by the hospital’s ethics committee, ensuring compliance with ethical standards.

Bacterial genomic DNA extraction was performed using the TIANamp Bacteria DNA Kit and DNA Purification Kit, both procured from TIANGEN Biotech (Shanghai, China). The RPA Basic Kit was supplied by TwistDx Co., Ltd. (Hertfordshire, United Kingdom). EnGen^®^ Lba Cas12a (Cpf1) and NEBuffer r2.1 were sourced from New England Biolabs (Guangzhou, China). The 2 × Es Taq MasterMix (Dye) for PCR was supplied by Cwbio Co. (Jiangsu, China). Nuclease-free water used in the experiments was obtained from Solarbio (China). Fluorescence data were recorded using the TILONG Gentier 96E/96R qPCR analysis system (Xi’an, China).

All RPA primers, PCR primers, crRNAs, and probes required for this study were synthesized by Songon Biotechnology Co. (Shanghai, China), and the cage modifications of crRNA were completed by Bio-lifesci Co. (Guangzhou, China).

### 2.2 Bacterial culture and extraction

Standard strains were preserved in glycerol at −80°C in an ultra-low temperature freezer. Before experimentation, bacterial suspensions were thawed at room temperature and cultured on blood agar plates, then incubated at 37°C with 20% CO_2_ for 24–48 h. Individual bacterial colonies (2–3 mm in diameter) were selected to prepare McFarland standard suspensions. Genomic DNA from standard strains was extracted using the TIANamp Bacteria DNA Kit. The concentration and purity of the extracted DNA were assessed with a spectrophotometer, and samples exhibiting OD260/280 ratios between 1.9 and 2.0 were considered suitable for subsequent analyses.

For clinical sputum specimens, pre-treatment with 4% NaOH was performed for at least 60 min to facilitate digestion. Following digestion, the samples were centrifuged at 12,000 rpm for 2 min. The supernatant was discarded, and the resulting pellet was processed using the TIANamp Bacteria DNA Kit to extract genomic DNA.

### 2.3 Design of primer and crRNA

Table 1 lists the primers, crRNA sequences, and fluorescent probe sequences involved in this study. Partial OXA-51 gene sequences of *A. baumannii* were retrieved from the NCBI database (http://www.ncbi.nlm.nih.gov/), and primers for OXA-51 were designed using an online primer design tool following the RPA Basic Kit instruction manual: primer length, 26–32 bp; amplicon size, 150–300 bp; GC content, 20%–70%; and a maximum allowable repeat length of 5 bases. The primer pair with the highest principle score was used as an alternative.

**TABLE 1:** Sequences involved in this study.

Name	Sequence (5′-3′)	Length(bp)
OXA-51-Forward primers	TAA​GGC​AAC​CAC​CAC​AGA​AGT​ATT​TAA​G	28
OXA-51-Reverse primers	CCT​CTT​GCT​GAG​GAG​TAA​TTT​TTA​AAG​G	28
crRNA	UAA​UUU​CUA​CUA​AGU​GUA​GAU​GCC​GCG​UAU​GGA​CUU​GA	38
Caged-crRNA	UAAUUUCUACUAAGUGUAGAUGC/NPOM-dt/CG/NPOM-dt/CGUAU/NPOM-dt/GGACUUGA	38
Fluorescent probe	FAM-TTATT-BHQ	5

**FIGURE 1:**
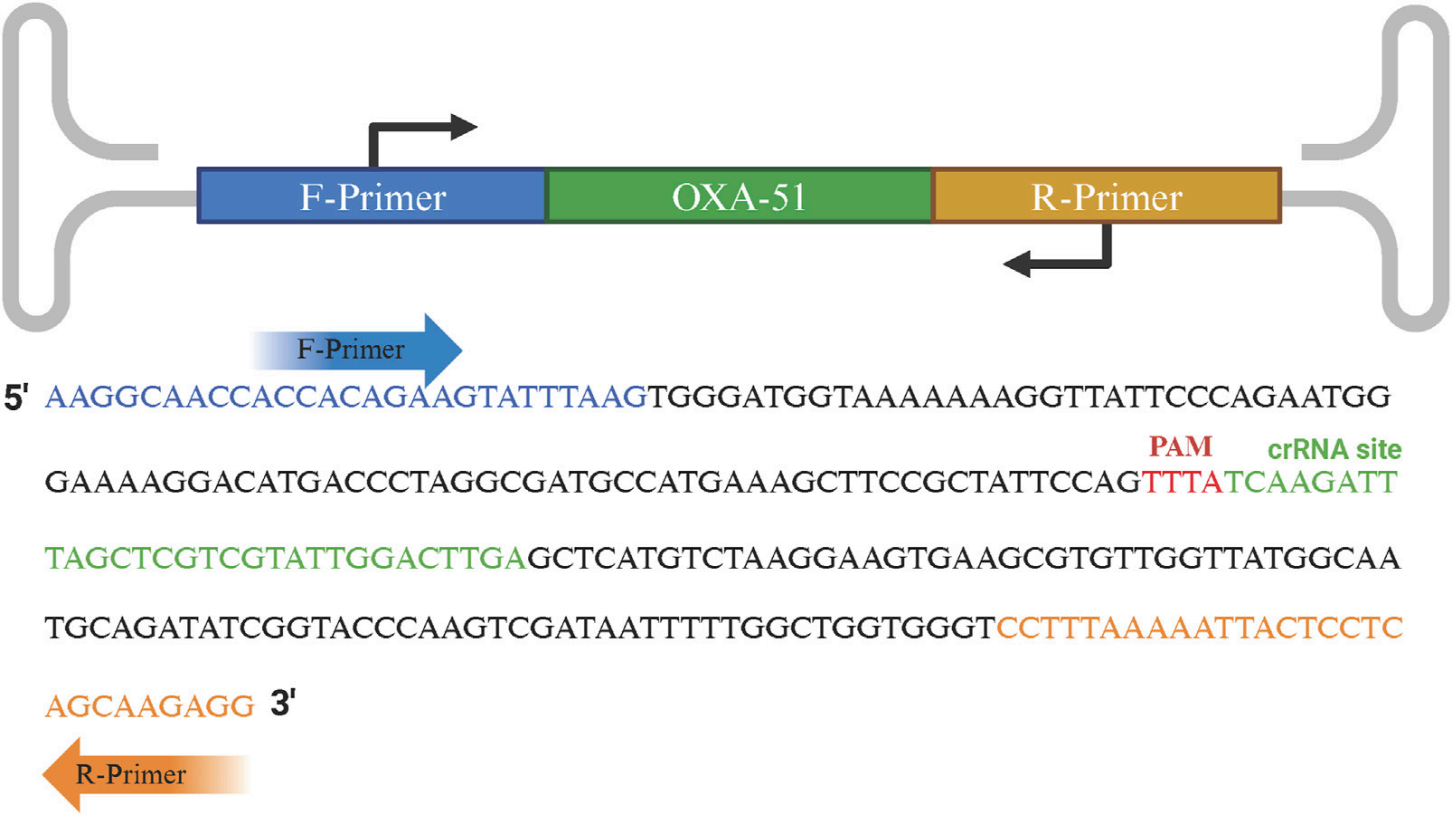
Schematic representation of RPA primers and crRNA recognition sequences. The forward and reverse primers are indicated in blue and orange text, respectively. The PAM site and crRNA recognition sequence are highlighted in red and green text, respectively.

The crRNA consists of two components: repeat and spacer. Within the amplified fragment, complementary sequences containing the protospacer adjacent motif (PAM, TTTN) were identified, and the 20 bp sequence immediately downstream of the PAM was selected as the target sequence. The repeat (5′-UAA​UUU​CUA​CUC​UUG​UAG​AU-3′) was then linked to the 5′ end of the spacer to form the specific crRNA. The amplified fragments, primers, and crRNA positions are shown in Figure 1.

The NCBI “BLAST” was used to evaluate the primer pair and crRNA sequence for conservation and specificity. Additionally, to generate caged crRNA, 3 NOPM-dt’s inserted into spacer sequence of crRNA (UAAUUUCUACUAAGUGUAGAUGC/NPOM-dt/CG/NPOM-dt/CGUAU/NPOM-dt/GGACUUGA). This modification ensured stability while allowing for selective deprotection under 365 nm UV light, without activation under other conditions.

### 2.4 Light-controlled one-tube RPA-CRISPR/Cas12a reaction system

In this diagnostic strategy, the RPA pre-amplification system and CRISPR-Cas12a system were combined into a single reaction mixture. Prior to the experiment, the lyophilized enzyme powder was dissolved in 29.5 μL of buffer solution according to the RPA instruction manual, and 2.4 μL each of forward and reverse primers (10 μM) along with 9.2 μL of nuclease-free water were added to prepare a pre-mix.

The 15 μL one-tube reaction system consisted of the following components: (a) 10 μL of RPA reaction mixture, including the lyophilized enzyme powder pre-mix, forward primer, reverse primer, template DNA, nuclease-free water, and Mg^2+^; (b) 5 μL of CRISPR/Cas12a reaction mixture, including 10 × r2.1 Buffer, Cas12a, caged crRNA, ssDNA, and nuclease-free water. The combined mixture was gently vortexed, briefly centrifuged, and incubated at 37°C for 20 min. Following the incubation, the reaction was exposed to UV light at a wavelength of 365 nm for 60 s to activate the caged crRNA. Fluorescence signals were then dynamically recorded every minute for 30 min at 37°C using the TIANLONG Gentier 96E/96R qPCR system, with FAM fluorescence signals collected in real-time.

### 2.5 Feasibility of light-controlled one-tube RPA-CRISPR/Cas12a reaction system

Previous studies have highlighted a significant challenge: the straightforward combination of RPA amplification with various CRISPR-based detection strategies in a one-tube format often results in significantly reduced detection efficiency (Arizti-Sanz et al., 2020). This is likely due to the excessive production of target fragments during the RPA pre-amplification phase, which simultaneously activates the CRISPR detection system. The cis-cleavage activity of Cas proteins is triggered, leading to continuous degradation of target fragments, which in turn hampers the trans-cleavage activity and diminishes overall detection performance. To address this issue, we utilized NPOM-dt to modify the crRNA within the CRISPR-Cas12a system, preventing the crRNA from base-pairing with the continuously generated target fragments. This modification renders the Cas12a/crRNA complex inactive, temporarily silencing the CRISPR-Cas12a system and effectively separating the pre-amplification and signal amplification processes on a temporal level.

To validate this approach, we designed a series of component omission experiments to evaluate the necessity of each component, including Cas12a protein, caged crRNA, uncaged crRNA, ssDNA probe, and template. Results were assessed through fluorescence signal intensity and agarose gel electrophoresis. Comparisons were made with a standard one-tube strategy utilizing uncaged crRNA to demonstrate the superiority of our approach. Furthermore, we investigated the stability of the caged crRNA under various lighting conditions, including natural light, incandescent light, infrared light, and LED light, to assess its robustness in diverse environments.

### 2.6 Optimization of reaction conditions

The reaction components in the light-controlled one-tube RPA-CRISPR/Cas12a detection system are complex and involve multiple biochemical reactions, making the concentrations of individual components critical to detection efficiency. In this section, we focus on optimizing the key components that influence reaction efficiency.

To determine the optimal primer concentration for the RPA pre-amplification phase, fluorescence values were measured across a concentration range of 200–440 nM at 80 nM intervals. Similarly, to establish the optimal reaction time for RPA pre-amplification, fluorescence values were recorded at time intervals ranging from 10 to 25 min in 5-min increments. Based on the combination that produced the highest fluorescence signal, the optimal RPA reaction conditions were determined.

To enhance the trans-cleavage activity of the CRISPR-Cas12a system, we further optimized a series of reaction conditions. First, gradient concentrations of Cas12a protein and caged crRNA were tested across a range of 100–420 nM at 80 nM intervals. Additionally, since the duration of UV light exposure directly impacts the deprotection efficiency and recovery rate of caged crRNA, the optimal exposure time was explored within a range of 10–90 s.

### 2.7 Sensitivity and specificity

To evaluate the sensitivity of the light-controlled one-tube RPA-CRISPR/Cas12a detection system, we used the PUC19-51 plasmid as the positive template. The initial concentration was 1 × 10^6^ copies/μL, which was serially diluted tenfold to a final concentration of 1 × 10^0^ copies/μL. This series of dilutions was used to validate the sensitivity of the detection system.

Subsequently, the specificity of the system was evaluated using six standard strains of clinically common pathogenic bacteria: *P. aeruginosa*, *E. coli*, *S. maltophilia*, *K. pneumoniae*, *S. aureus*, and *H. haemolyticus*. Fluorescence intensity results were used to provide a clear assessment of the system’s specificity. Detailed information regarding the bacterial strains is summarized in Table 2.

**TABLE 2:** Source of bacterial strains.

Bacteria	Source
*A. baumannii*	ATCC 19606
*E*. *coli*	ATCC 25922
*S*. *aureus*	ATCC 25923
*K*. *pneumoniae*	ATCC 700603
*P*. *aeruginosa*	ATCC 27853
*S*. *maltophilia*	ATCC 19861
*H. haemolyticus*	ATCC 33390

### 2.8 Detection of clinical sputum samples

A total of 38 clinical sputum samples were collected from the Microbiology Laboratory of the Affiliated Hospital of Youjiang Medical University for Nationalities to evaluate the clinical performance of the light-controlled one-tube RPA-CRISPR/Cas12a detection system. The samples were assigned identification numbers S1-S38. Among these, S3, S4, S14, S15, S25, S32, S33, and S38 were identified as negative for *Acinetobacter* baumannii using culture-based methods and mass spectrometry, while the remaining samples were confirmed as positive.

Two professional technicians from the same laboratory, blinded to the sample statuses, participated in the experiment. One technician tested the samples using the light-controlled one-tube RPA-CRISPR/Cas12a method, while the other used conventional PCR. The results of the two methods were compared to assess their consistency.

The PCR reaction was prepared in a total volume of 25 μL, comprising 12.5 μL of 2 × Es Taq MasterMix (Dye), 1.25 μL of forward primer, 1.25 μL of reverse primer, 1 μL of DNA template, and 9 μL of ddH_2_O. The thermal cycling conditions were as follows: an initial denaturation at 94°C for 2 min, followed by 35 cycles of 94°C for 10 s, 53°C for 30 s, and 72°C for 30 s, with a final extension at 72°C for 2 min. The PCR products were analyzed using agarose gel electrophoresis for visualization.

### 2.9 Statistical analysis

Statistical analyses were conducted using GraphPad Prism version 9.5 (GraphPad Software Inc., San Diego, CA, United States). A two-way analysis of variance (ANOVA; mixed-effects model) was employed for statistical evaluation. Data are expressed as mean ± standard deviation (SD). Statistically significant differences relative to the control group were identified, with results based on three technical replicates (n = 3). Statistical significance levels were denoted as follows: ****P < 0.0001, ***P < 0.001, **P < 0.01, and *P < 0.05. SD refers to the standard deviation of the mean.

## 3 Result

### 3.1 Development and validation of a light-controlled one-tube RPA-CRISPR/Cas12a detection system

**FIGURE 2:**
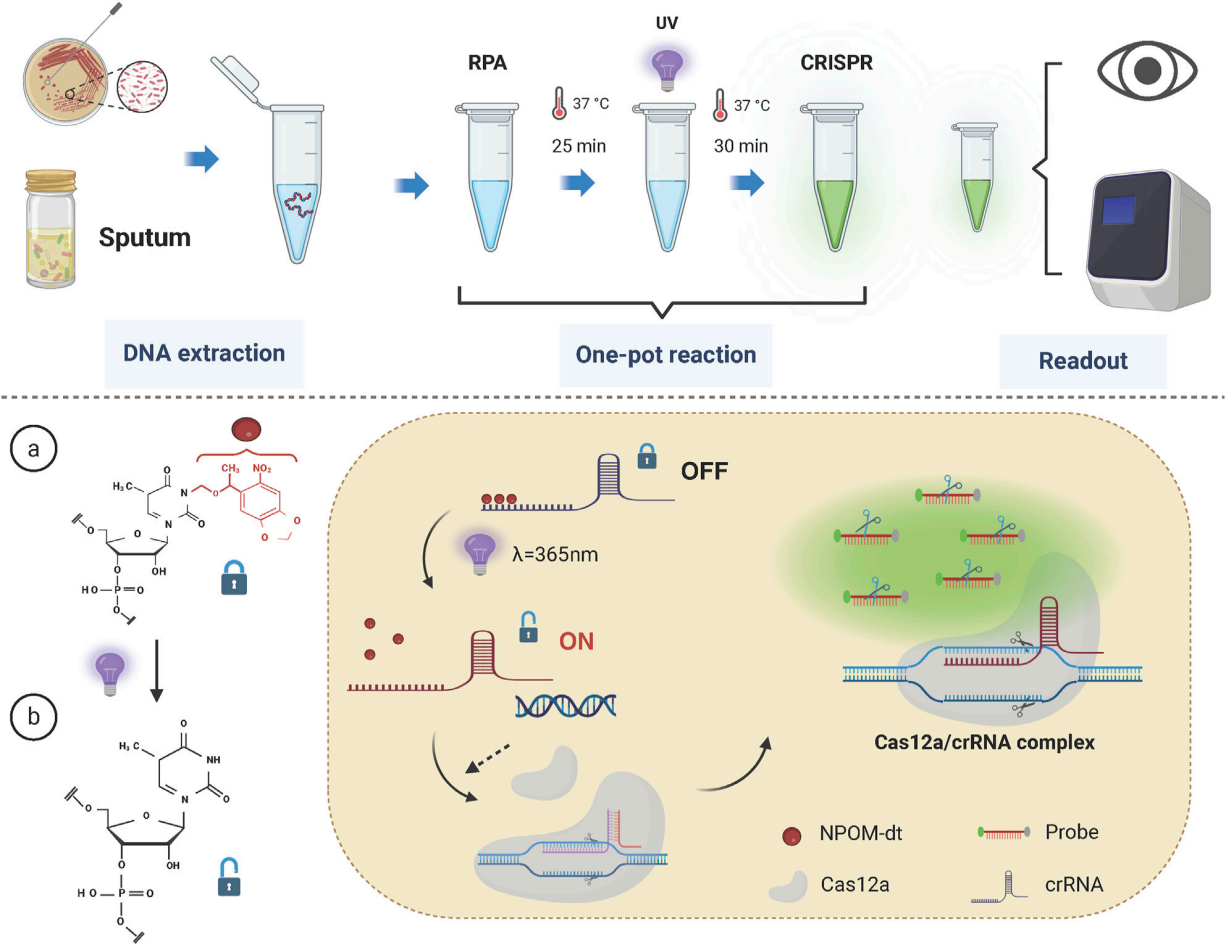
Schematic of the light-controlled one-tube RPA-CRISPR/Cas12a system workflow. The RPA pre-amplification and CRISPR/Cas12a system are integrated into a single reaction tube. Upon completion of RPA pre-amplification, the reaction mixture is exposed to UV light at a wavelength of 365 nm, triggering the release of crRNA from its cage and restoring the activity of the CRISPR/Cas12a system.

In this study, primers and crRNA targeting the intrinsic OXA-51 gene of *Acinetobacter* baumannii were designed, and the crRNA was modified using the NPOM-dt chemical group. A light-controlled one-tube system combining RPA with CRISPR-Cas12a was developed to facilitate the detection of *A. baumannii* infections. An overview of the detection workflow and reaction mechanism is provided in Figure 2.

In the one-tube system, RPA is employed for pre-amplification and is integrated with CRISPR/Cas12a-mediated detection. The key innovation of this strategy lies in the use of NPOM-dt-modified crRNA. Upon brief exposure to UV light at a wavelength of 365 nm, the nucleoside modification is rapidly degraded, restoring the crRNA’s ability to base-pair with the target sequence. Once sufficient target fragments with PAM sites are generated during the RPA pre-amplification phase, UV light activation restores the activity of the crRNA. Subsequently, the Cas12a protein binds with the crRNA to form the Cas12a/crRNA complex, which then interacts with the target fragments to form a ternary complex, activating the Cas12a protein.

Activated Cas12a exhibits trans-cleavage activity, resulting in the indiscriminate cleavage of surrounding single-stranded DNA probes. The CRISPR/Cas12a system-guided nucleic acid cleavage operates efficiently at temperatures around 37°C, which coincides with the optimal working temperature of RPA (Munawar, 2022; James and Macdonald, 2015; Williams et al., 2019). Therefore, the developed one-tube reaction strategy can be executed within a single tube and at a constant temperature, significantly reducing operational complexity and the risk of cross-contamination, while streamlining the detection workflow.

### 3.2 Feasibility of light-controlled one-tube RPA-CRISPR/Cas12a reaction system

**FIGURE 3:**
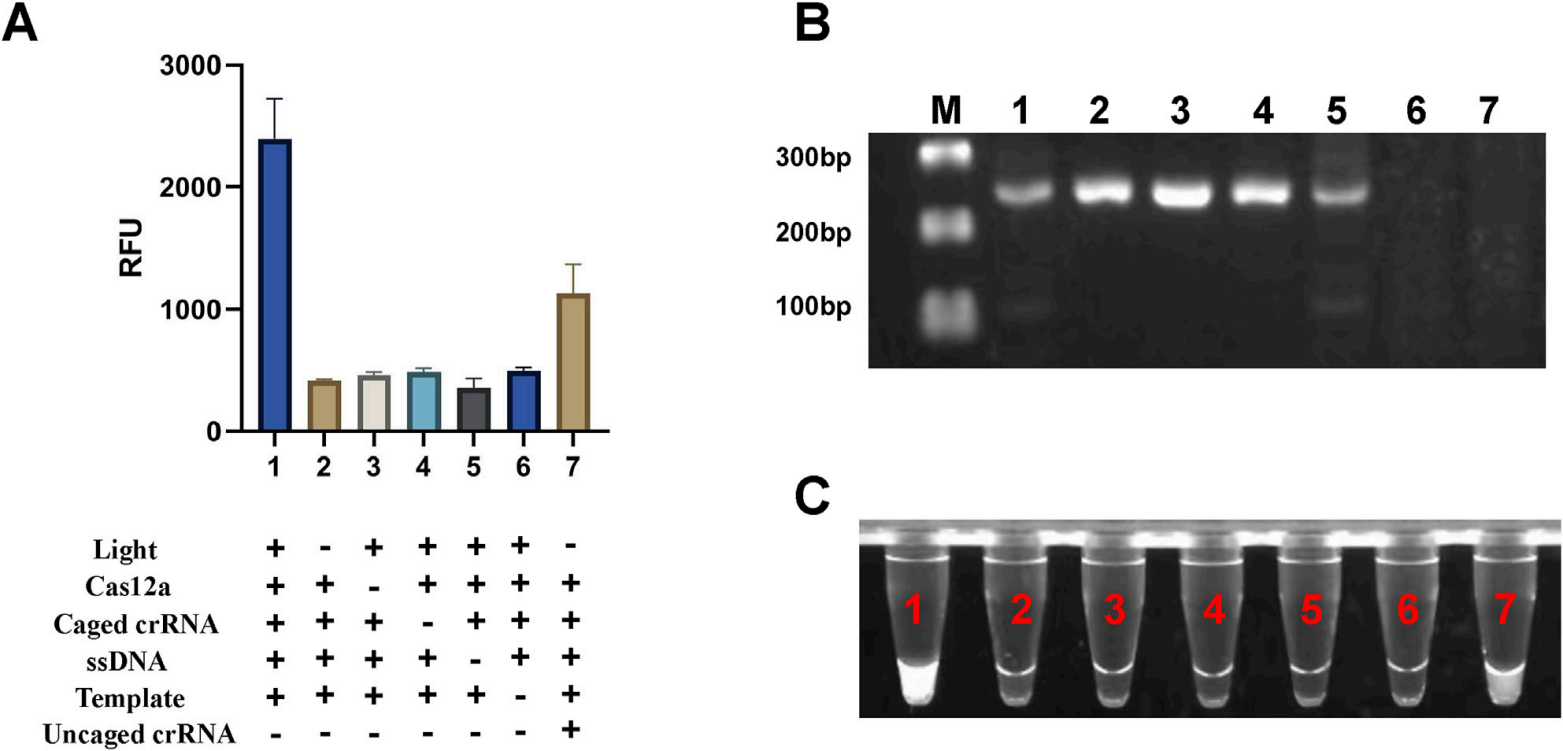
Validation of the light-controlled one-tube RPA-CRISPR/Cas12a system. **(A)** Fluorescence values from component omission experiments for proof-of-concept verification of the light-controlled one-tube RPA-CRISPR/Cas12a system. **(B)** Corresponding gel electrophoresis results. **(C)** UV fluorescence images of the light-controlled one-pot RPA-CRISPR/Cas12a system. Labels 1-7 indicate different experimental groups. M, Marker.

**FIGURE 4:**
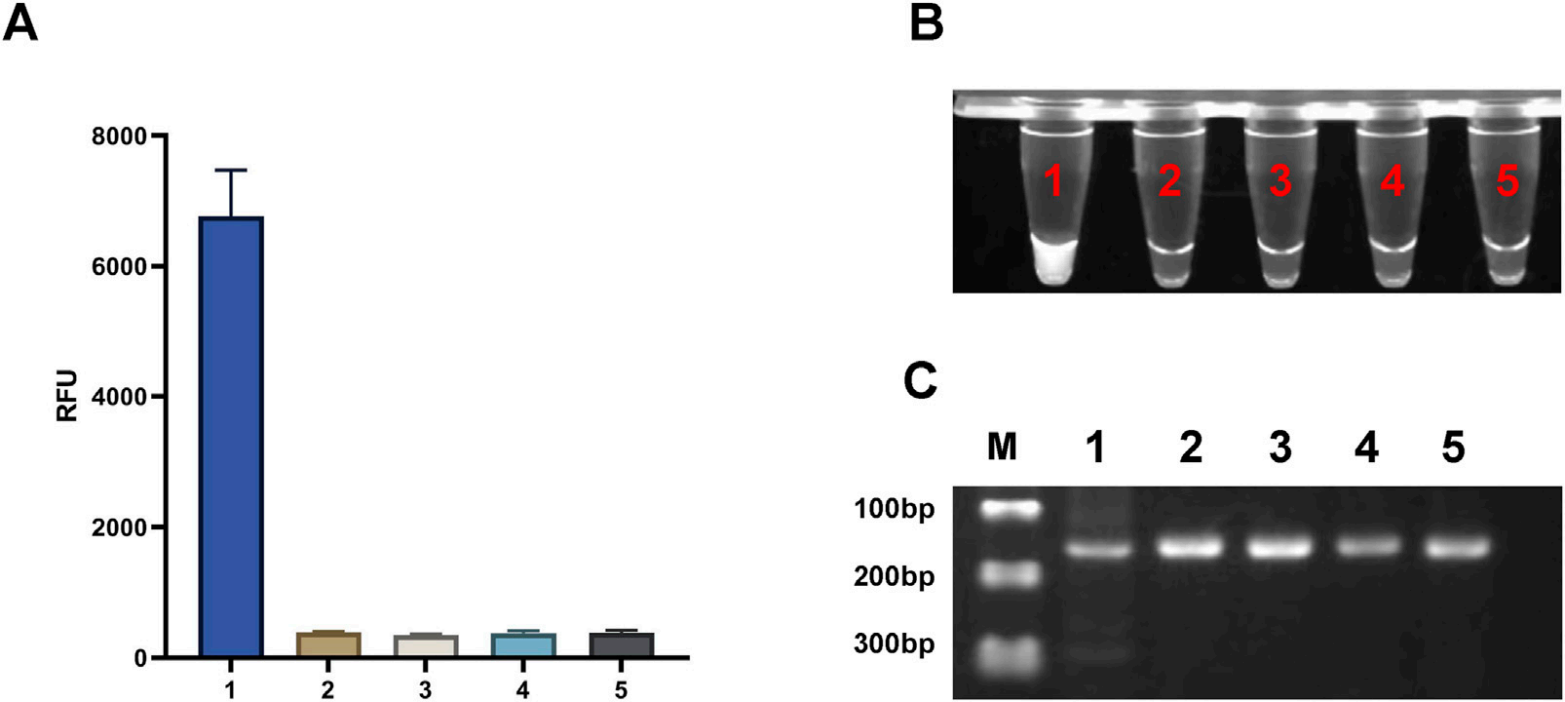
Effects of Different Light Sources on the light-controlled one-tube RPA-CRISPR/Cas12a system. **(A)** Fluorescence intensity under different illumination conditions. ****P < 0.0001 (n = 3). **(B)** UV fluorescence images of the one-tube RPA-CRISPR/Cas12a system. **(C)** Gel electrophoresis results of the one-tube RPA-CRISPR/Cas12a system. Labels 1–5 correspond to UV light, natural light, LED light, incandescent light, and darkroom conditions, respectively. M, Marker.

To validate the effectiveness of the light-controlled one-pot detection method, we designed component omission experiments. In the six experimental groups, specific fluorescence signals were observed only when all required components and environmental conditions were present (Figures 3A, C, NO.1). In contrast, the absence of any single component prevented the initiation of specific signal amplification (Figures 3A, C, NO.2-NO.6). Agarose gel electrophoresis results further confirmed that trans-cleavage activity was activated only under UV light exposure and in the presence of essential components, including Cas12a, crRNA, and the template DNA (Figure 3B). We also conducted a parallel comparison of the efficiency of caged versus uncaged crRNA in the one-tube detection system using PUC19-51 plasmid at the same concentration as the template. The light-controlled one-tube method with caged crRNA demonstrated a significantly enhanced fluorescence signal. In contrast, the uncaged crRNA system showed a marked reduction in fluorescence efficiency, likely due to the extensive depletion of the template prior to CRISPR detection (Figures 3A, C, NO.7; Figure 3B, Lane 7). In addition, we irradiated the caged crRNA under different light conditions to evaluate whether it would non-specifically trigger the decannation reaction under different light conditions, and the results showed that only the caged crRNA underwent decannation under UV light, which activated the CRISPR/Cas12a system and cleavaged the amplicon. In contrast, no amplicon cleavage was observed under other lighting conditions, indicating that the caged crRNA did not undergo a cage-out reaction (Figure 4).

### 3.3 Optimization of reaction conditions

**FIGURE 5:**
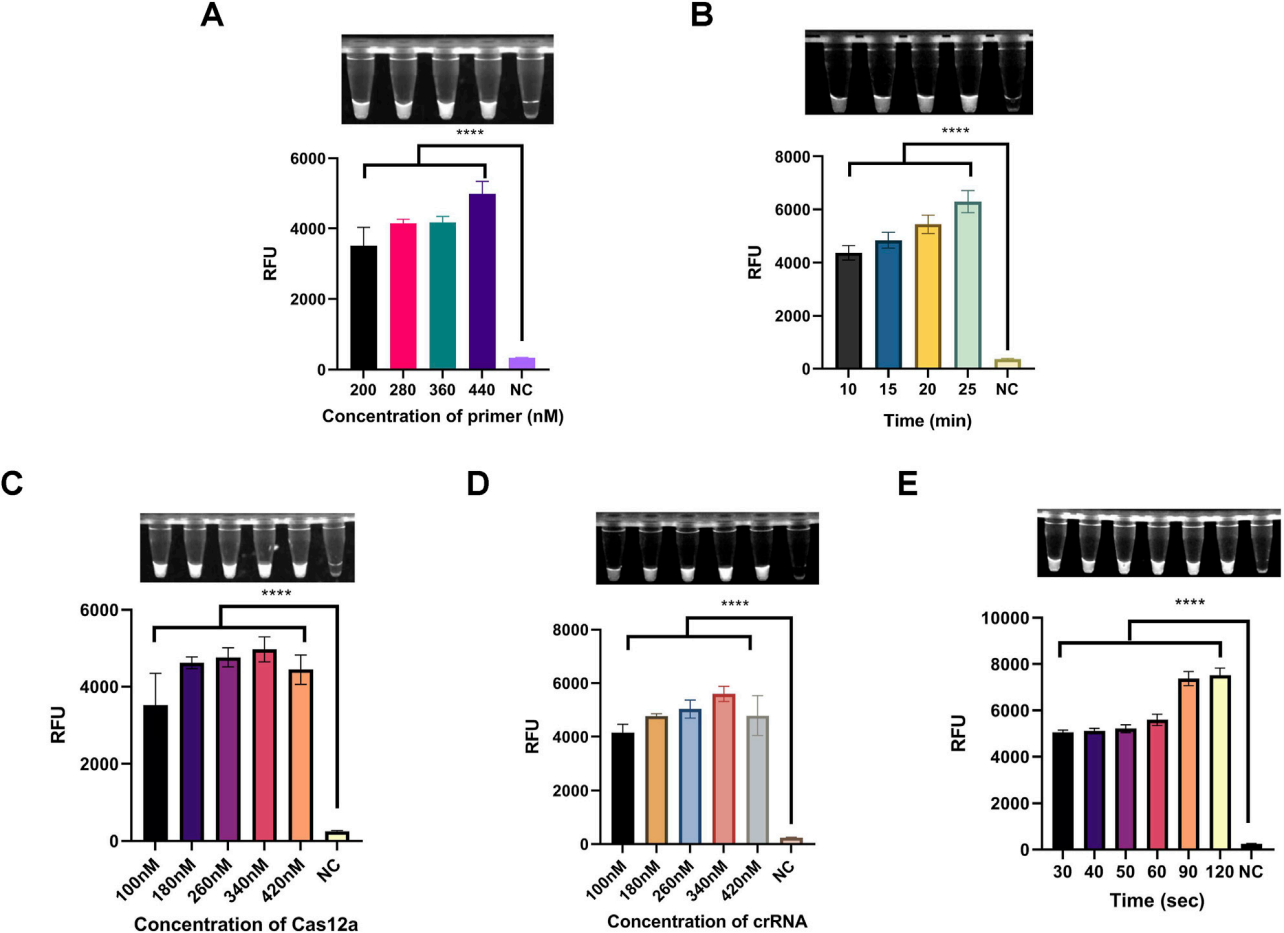
Optimization of reaction conditions for the light-controlled one-tube RPA-CRISPR/Cas12a system. **(A)** Optimization of primer concentration. **(B)** Optimization of RPA pre-amplification duration. **(C)** Optimization of Cas12a concentration. **(D)** Optimization of caged crRNA concentration. **(E)** Optimization of UV exposure duration. ****P < 0.0001 (n = 3).

Primer concentration and amplification duration are critical factors influencing amplification efficiency. As shown in Figure 5A, the one-tube system demonstrated optimal fluorescence intensity across primer concentrations ranging from 200–440 nM, with 440 nM identified as the optimal concentration. Additionally, fluorescence intensity increased with amplification times ranging from 10 to 25 min (Figure 5B). Considering the balance between efficiency and time, 25 min was selected as the optimal amplification duration.

Next, to determine the optimal concentration ratio of Cas12a to crRNA, a gradient range of 100–420 nM was tested. As shown in Figures 5C,D, fluorescence intensity reached its peak at a concentration of 340 nM for both Cas12a protein and crRNA. Therefore, 340 nM was selected as the optimal concentration for these components.

The duration of UV exposure significantly affects the deprotection efficiency of NPOM-dt. We investigated exposure times ranging from 30–120 s to assess their impact on the one-tube system. The results indicated that fluorescence intensity plateaued at 90 s (Figure 5E). Thus, 90 s was determined to be the optimal UV exposure duration.

### 3.4 Sensitivity and specificity

**FIGURE 6:**
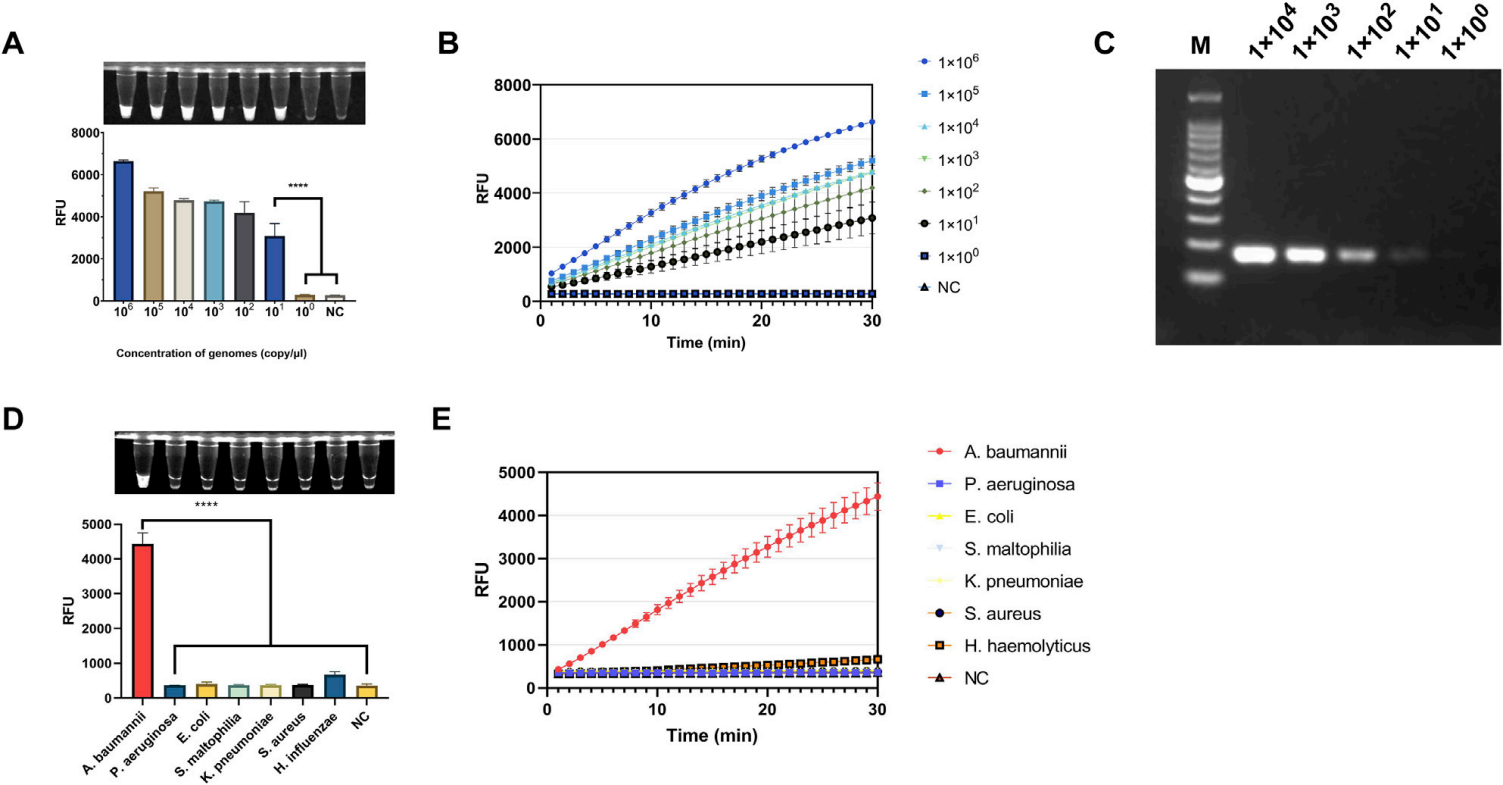
Evaluation of the Sensitivity and Specificity of the light-controlled one-tube RPA-CRISPR/Cas12a system. **(A)** Endpoint fluorescence values for the determination of the detection limit using PUC19-51 plasmid standards. **(B)** Real-time fluorescence curves for detection limit determination. **(C)** Sensitivity of PCR for plasmid detection (copies/μL). **(D)** Endpoint fluorescence values for specificity evaluation using genomic DNA from six clinically relevant pathogenic bacteria. **(E)** Real-time fluorescence curves for specificity evaluation. ****P < 0.0001 (n = 3).

The PUC19-51 recombinant plasmid was serially diluted to concentrations ranging from 1 × 10^6^ to 1 × 10^0^ copies/μL and used as a template to validate the light-controlled one-tube RPA-CRISPR-Cas12a system. Fluorescence results demonstrated the ability to detect the OXA-51 gene at a minimum concentration of 1 × 10^1^ copies/μL. (Figures 6A,B). Additionally, we found that the sensitivity of the light-controlled one-tube strategy was comparable to that of PCR (Figure 6B). For the specificity validation, genomic DNA extracted from the standard strain of *Acinetobacter baumannii* (ATCC19606) and six other clinical pathogens as templates, and only the genomic DNA of *A. baumannii* showed significant fluorescence intensity (Figures 6C,D).

### 3.5 Detection of clinical samples

**FIGURE 7:**
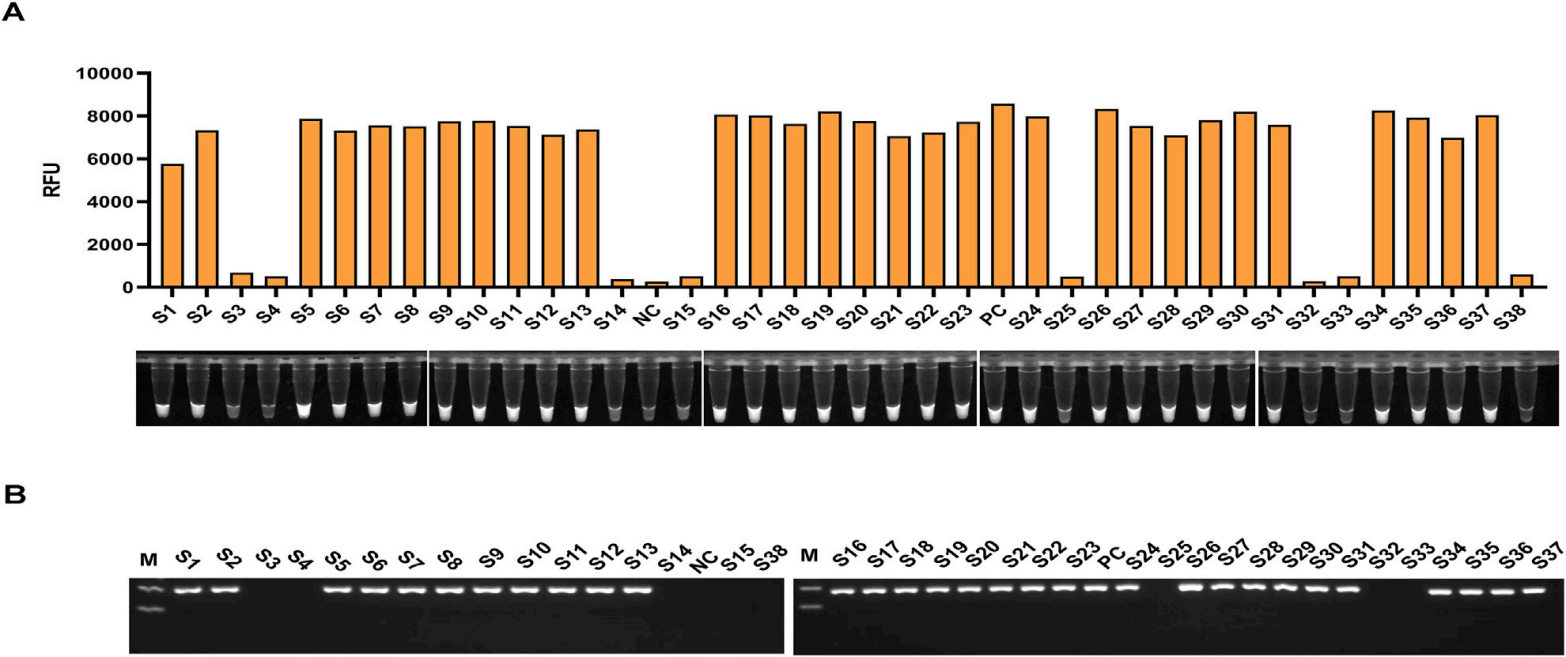
Clinical Validation of the light-controlled one-tube RPA-CRISPR/Cas12a system. The infection status of 38 clinical sputum samples was confirmed using culture methods. A double-blind approach was employed to perform parallel testing of all clinical samples using **(A)** the light-controlled one-tube RPA-CRISPR/Cas12a system and **(B)** conventional PCR. Samples S1–S38 represent sputum specimen IDs, with S3, S4, S14, S15, S25, S32, S33, and S38 identified as uninfected with *A. baumannii*.

The clinical performance of the light-controlled one-tube RPA-CRISPR/Cas12a detection system was evaluated using clinical sputum samples. PCR results showed that the OXA-51 gene was successfully amplified in 30 clinical samples, confirming *Acinetobacter* baumannii infection in these cases, while 8 samples were negative. The detection results of the light-controlled one-tube RPA-CRISPR/Cas12a system were fully consistent with those of PCR (Figure 7; Table 3).

**TABLE 3:** Detection results of Light-controlled one-tube RPA-CRISPR/Cas12a and PCR.

Light-controlled one-tube RPA-CRISPR/Cas12a	PCR		Total
	Positive	Negative	
Positive	30	0	30
Negative	0	8	8
Total	30	8	38

These findings demonstrate that the light-controlled one-tube RPA-CRISPR-Cas12a detection system and PCR method exhibit equivalent clinical performance. This suggests that the developed system has the potential to serve as an alternative to PCR for detecting *A. baumannii* infections.

## 4 Discussion

*A. baumannii* is a common pathogen responsible for ventilator-associated pneumonia and frequently causes secondary invasive infections such as meningitis, urinary tract infections, surgical site infections, and sepsis. Carbapenem antibiotics are the first-line treatment for multidrug-resistant *A. baumannii* infections. However, with the widespread and often inappropriate use of antibiotics, carbapenem resistance has become increasingly prevalent, leading to the emergence of pan-drug-resistant (PDR) strains. This has become a critical global public health issue, as treatment options for PDR *A. baumannii* infections remain extremely limited. Consequently, effective and timely prevention strategies are essential for controlling the spread of infections. There is an urgent need for diagnostic tools that are simple, cost-effective, sensitive, and suitable for use in community hospitals or even for at-home testing. These tools must enable pathogen detection during the infection window period to prevent large-scale public health outbreaks.

The CRISPR system relies on crRNA to guide the formation of complexes of the Cas protein, which recognizes the target and activates the Cas protein through base pairing. The activated Cas protein has both cis (specific cleavage target) and trans (indiscriminately cleaves nearby single-stranded DNA probes to amplify the signal) cleavage activity, making it a powerful tool for molecular diagnostics. It is highly compatible with isothermal amplification technology, which has been attracting attention in recent years. This combination simplifies operations while significantly improving sensitivity and specificity, providing a new approach to POCT.

However, most current RPA-CRISPR detection strategies adopt a two-step workflow. In these protocols, RPA amplification is performed in one tube, and the amplified product is subsequently transferred to another tube for CRISPR detection. This approach is cumbersome, increases the risk of aerosol contamination, and lacks the simplicity of single-tube PCR workflows. Colorimetric sensing technology and electrochemical sensing technology based on nanoenzymes have been widely used in the diagnostic field in recent years, with the advantages of fast response speed, wide applicability, and lower equipment and reagent requirements (Qiu et al., 2023; Uno et al., 2023; Tan et al., 2024).

Some researchers have proposed methods such as using gel, paraffin, and mother and child tubes for physical separation, or directly adding the CRISPR system to the test tube cap to achieve single-tube operation, which are relatively stable and achieve one-tube detection at the spatial level. However, the above methods still require steps such as temperature adjustment or manual centrifugation to mix the two systems with each other, and the labor cost is still high (Yu et al., 2024; Wen et al., 2024; Liu et al., 2025).

To achieve rapid detection of *A. baumannii* during the infection window and to control infection rates, we developed a light-controlled, one-tube RPA-CRISPR/Cas12a detection system. This method was optimized for pre-amplification duration, UV exposure time, and reaction component concentrations, enabling the detection of *A. baumannii* within 60 min. The system integrates RPA-based target amplification with CRISPR-mediated signal amplification into a single tube, significantly simplifying operational steps and reducing procedural complexity.

In this system, the crRNA is chemically modified with NPOM-dt groups to temporarily block its ability to base-pair with target fragments, rendering the CRISPR system inactive. After RPA generates sufficient target fragments, UV light exposure induces the deprotection of the NPOM-dt group, restoring crRNA activity and activating the CRISPR/Cas12a system. This light-controlled, one-tube detection strategy avoids the need for repetitive tube opening and minimizes aerosol contamination risks. More over, the light-controlled one-tube RPA-CRISPR/Cas12a system simplifies reagent design, optimizes single-tube operation and other innovations, and achieves breakthroughs in timeliness, economy, and applicability, with reagent costs as low as $5/test and can be completed within 60 min, and the equipment cost only relies on a constant temperature heater and ultraviolet light source, which provides an efficient and low-cost technical path for nucleic acid diagnosis in POCT and resource-constrained scenarios.

Despite its promising performance, the light-controlled one-tube detection strategy has certain limitations. First, the clinical sample size of this study is small and the sample type is single (only sputum samples are collected), and we will continue to expand the sample size and collect samples from different sources for clinical performance verification in the future. Secondly, the combination of RPA and CRISPR involves multiple biochemical components, including various enzymes, DNA, and RNA, which may create molecular crowding effects and lead to inconsistent results. At last, due to current laboratory constraints, the method requires manual UV exposure, preventing fully automated operation. Future research should focus on developing an integrated device that incorporates heating, mixing, UV light exposure, and real-time fluorescence monitoring. Such a portable system would make the method more suitable for at-home testing and point-of-care diagnostics. With further optimization, this strategy has the potential to become a highly efficient, cost-effective, and user-friendly molecular diagnostic tool for preventing and controlling public health emergencies.

## 5 Conclusion

In summary, we have developed a light-controlled, one-pot RPA-CRISPR/Cas12a detection system for the rapid and sensitive identification of *A. baumannii*. This system operates under isothermal conditions (37°C) and can detect target fragments at concentrations as low as 10 copies/μL, with no cross-reactivity observed with six other clinically common pathogenic bacteria. The key advantages of this detection system lie in its low cost, minimal equipment requirements, and the elimination of aerosol contamination risks associated with repeated tube opening and transfer. Additionally, its simplified workflow makes it particularly suitable for resource-limited settings, offering a novel approach to advancing point-of-care diagnostics and immediate testing solutions.

## Data Availability

The original contributions presented in the study are included in the article/supplementary material, further inquiries can be directed to the corresponding authors.
